# FAHD1 and mitochondrial metabolism: a decade of pioneering discoveries

**DOI:** 10.1111/febs.17345

**Published:** 2024-12-06

**Authors:** Elia Cappuccio, Max Holzknecht, Michèle Petit, Anne Heberle, Yana Rytchenko, Athanasios Seretis, Ciro L. Pierri, Hubert Gstach, Pidder Jansen‐Dürr, Alexander K. H. Weiss

**Affiliations:** ^1^ Faculty of Biology, Institute for Biomedical Aging Research Universität Innsbruck Austria; ^2^ Department of Pharmacy‐Pharmaceutical Sciences Università di Bari Italy; ^3^ Department of Pharmaceutical Sciences, Faculty of Life Sciences University of Vienna Austria

**Keywords:** aging and cellular senescence, cancer metabolism, FAHD1, glutamine metabolism, mitochondrial dysfunction, mitochondrial metabolism, ODx, ROS, TCA cycle

## Abstract

This review consolidates a decade of research on *fumarylacetoacetate hydrolase domain containing protein 1* (FAHD1), a mitochondrial oxaloacetate tautomerase and decarboxylase with profound implications in cellular metabolism. Despite its critical role as a regulator in mitochondrial metabolism, FAHD1 has remained an often‐overlooked enzyme in broader discussions of mitochondrial function. After more than 12 years of research, it is increasingly clear that FAHD1's contributions to cellular metabolism, oxidative stress regulation, and disease processes such as cancer and aging warrant recognition in both textbooks and comprehensive reviews. The review delves into the broader implications of FAHD1 in mitochondrial function, emphasizing its roles in mitigating *reactive oxygen species* (ROS) levels and regulating complex II activity, particularly in cancer cells. This enzyme's significance is further highlighted in the context of aging, where FAHD1's activity has been shown to influence cellular senescence, mitochondrial quality control, and the aging process. Moreover, FAHD1's involvement in glutamine metabolism and its impact on cancer cell proliferation, particularly in aggressive breast cancer subtypes, underscores its potential as a therapeutic target. In addition to providing a comprehensive account of FAHD1's biochemical properties and structural insights, the review integrates emerging hypotheses regarding its role in metabolic reprogramming, immune regulation, and mitochondrial dynamics. By establishing a detailed understanding of FAHD1's physiological roles and therapeutic potential, this work advocates for FAHD1's recognition in foundational texts and resources, marking a pivotal step in its integration into mainstream metabolic research and clinical applications in treating metabolic disorders, cancer, and age‐related diseases.

## Graphical abstract

This paper reviews a decade of research on FAHD1, an important yet underappreciated enzyme in mitochondrial metabolism. FAHD1 plays a crucial role in energy production, oxidative stress regulation, and processes related to aging and overall health. Here, we highlight FAHD1's potential as a therapeutic target due to its influence on key metabolic pathways essential for cellular function. The research advocates for broader recognition of FAHD1 in metabolic science, especially regarding its relevance to aging and metabolic health. The mitochondrial schematic in the background of the Graphical Abstract was generated using DALL‐E, an AI‐based image generation tool.

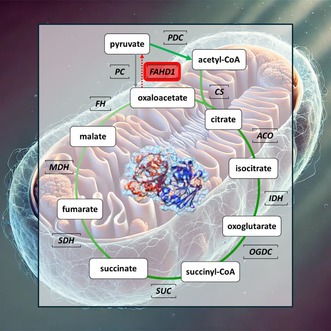

**Abbreviations**
AAT: aspartate aminotransferase
AAT/SGPT: aspartate aminotransferase
ACACA: acetyl‐CoA carboxylase
ACLY: ATP citrate lyase
AGAT: glycine amidinotransferase
ARG1: arginase 1
ASL: argininosuccinate lyase
ASS: argininosuccinate synthase
AST: aspartate transaminase
AST/SGOT: aspartate transaminase
ATP: adenosine triphosphate
CAC: carnitine/acylcarnitine carrier
CACT: carnitine/acylcarnitine translocase
CPT1, CPT2: carnitine palmitoyltransferase 1/2
FAHD1: fumarylacetoacetate hydrolase domain containing protein 1
FASN: fatty acid synthase
GOT1, GOT2: glutamate‐oxaloacetate transaminases
GSH: glutathione
MDH: malate dehydrogenase
mtDNA: mitochondrial DANN
OAA: oxaloacetate
ODx: oxaloacetate decarboxylase
OGC: oxoglutarate carrier
OXPHOS: oxidative phosphorylation
PEP: phosphoenolpyruvate
PEPCK: phosphoenolpyruvate carboxykinase
PYR: pyruvate
ROS: reactive oxygen species
SASP: senescence‐associated secretory phenotype
SLC25A1: citrate carrier
SLC25A10: dicarboxylate carrier (DIC)
SLC25A11: oxoglutarate carrier (OGC)
SLC25A12: aspartate/glutamate carrier (AGC1)
SLC25A13: aspartate/glutamate carrier (AGC2)
SLC25A15: ornithine carrier
SLC25A20: carnitine/acylcarnitine carrier/translocase complex
SLC25A23, SLC25A24, SLC25A25, SLC25A41: ATP‐Mg/Pi carriers
SLC25A31: ADP/ATP carrier
SLC25A4: ADP/ATP carrier
SLC25A5: ADP/ATP carrier
SLC25A6: ADP/ATP carrier
SLC25A7, SLC25A8, SLC25A9: uncoupling proteins (UCPs)
TCA: tricarboxylic acid cycle

## Introduction

The mitochondrial enzyme *fumarylacetoacetate hydrolase domain containing protein 1* (FAHD1) has become an increasingly studied target within mitochondrial biology and metabolic research. While it may still be lesser known in broader research circles, FAHD1's bifunctional activities [1, 2] are proving to be significant in mitochondrial metabolism [3]. Its role in regulating the *tricarboxylic acid* (TCA) cycle is gaining recognition, particularly among researchers investigating mitochondrial function and dysfunction [4, 5]. Growing evidence from several research groups underscores the enzyme's importance, and its involvement in various pathological contexts continues to attract attention.

Initial discoveries delved into the structural and functional nuances of FAHD1 [5, 6, 7, 8]. FAHD1 has been implicated in regulating complex II activity in cancer cells, underscoring its broader impact in cellular metabolism and disease states [9]. Notably, the enzyme's activity appears to be crucial for the survival of certain breast cancer cell lines, highlighting its potential as a target for cancer therapy. Subsequent research highlighted FAHD1's involvement in critical cellular processes, including its role in mitigating oxidative stress by reducing *reactive oxygen species* (ROS) levels in osteosarcoma cells [8, 10].

Studies using high‐resolution X‐ray crystallography provided insights into the enzyme's substrate processing and potential inhibition mechanisms, revealing how FAHD1 interacts with oxaloacetate and influences metabolic flux [11, 12]. This structural understanding has paved the way for developing inhibitors that could modulate FAHD1 activity, presenting therapeutic possibilities for conditions associated with mitochondrial dysfunction [3, 9].

This review aims to consolidate previous findings on FAHD1, integrating insights in the TCA cycle and mitochondrial metabolism. This collaborative effort provides a detailed account of FAHD1's biochemical properties, physiological roles, and therapeutic potential. By combining expert insights, this work strives to offer a comprehensive resource on FAHD1, enhancing the understanding and application of this important mitochondrial enzyme.

## Mitochondria in health, aging, and disease

Mitochondria play a pivotal role in cellular metabolism [13]. They serve as the metabolic hub within cells, orchestrating the processes of energy production, substrate metabolism, and cellular signaling [13, 14, 15, 16, 17, 18]. Understanding the intricate relationship between mitochondria and metabolism is not only fundamental to basic cell biology but also holds significant implications for developing therapeutic strategies to address various diseases associated with mitochondrial dysfunction. Mitochondria participate in the metabolism of various substrates, such as carbohydrates, amino acids, and lipids, through interconnected metabolic pathways. They influence cell survival by playing a critical role in apoptosis, the programmed cell death essential for maintaining tissue integrity, and eliminating damaged cells [13]. Their multifaceted roles highlight the importance of these organelles in maintaining cellular health and function. Mitochondria are dynamic organelles capable of fission and fusion, enabling them to respond to changing cellular conditions [19]. The removal of heavily damaged mitochondria through a process called mitophagy ensures the maintenance of a healthy mitochondrial population [20, 21, 22]. Dysregulation of mitochondrial metabolism is implicated in various diseases, including metabolic disorders, neurodegenerative diseases, and cancer, emphasizing the fundamental role these organelles play in maintaining cell fate, cellular homeostasis, and overall health [13, 14, 23, 24, 25].

One of the key functions of mitochondria is to generate *adenosine triphosphate* (ATP), the primary energy currency of the cell [13]. Through *oxidative phosphorylation* (OXPHOS), mitochondria utilize the energy derived from the breakdown of nutrients, such as carbohydrates, lipids, and amino acids, to pump protons across their inner mitochondrial membrane. This creates an electrochemical gradient that powers the ATP synthase enzyme, ultimately leading to the production of ATP. This tightly regulated process ensures that cells have a constant and reliable source of energy to carry out essential functions. Beyond ATP production, mitochondria are deeply engaged in maintaining cellular homeostasis [13, 14, 24].

Mitochondria play a critical role in cellular senescence, a state of irreversible cell cycle arrest that is associated with aging [24, 26, 27, 28, 29]. The mitochondrial theory of aging proposes that accumulated damage to mitochondria over time contributes to the aging process [27]. As cells undergo repeated cycles of energy production through OXPHOS, mitochondria generate ROS as byproducts [30, 31, 32]. Persistent oxidative stress causes damage to mitochondrial DNA, proteins, and lipids. Accumulation of damaged macromolecules, combined with impaired mitochondrial function, is thought to contribute to cellular dysfunction and the aging phenotype [13, 24, 28]. Senescent cells exhibit altered mitochondrial dynamics, including fragmented morphology and dysfunction [24]. In addition, accumulation of *mitochondrial DNA* (mtDNA) damage is a prominent feature associated with aging [33, 34]. During aging, the mitochondria are susceptible to various sources of damage, including ROS generated during normal cellular metabolism. The gradual accumulation of mutations and lesions in mtDNA contributes to mitochondrial dysfunction and has been implicated in aging. The release of pro‐inflammatory and other potentially harmful molecules, including extracellular vesicles, from senescent cells, collectively known as the *senescence‐associated secretory phenotype* (SASP), can further contribute to tissue inflammation and age‐related pathologies [13, 24, 28, 29]. Thus, the interplay between mitochondrial function and cellular senescence is a key aspect of aging biology [26]. It was shown that mitochondria are required for pro‐aging features of the senescent phenotype [35].

Beyond energy production and senescence, mitochondria are central to the regulation of apoptosis, a form of programmed cell death [13, 14, 25]. Dysfunctional mitochondria release factors such as cytochrome c that can promote cell death and/or contribute to chronic low‐grade inflammation, both of which are associated with aging and age‐related diseases [13, 24]. Mitochondrial dysfunction is implicated in various age‐related disorders, including neurodegenerative, cardiovascular, and metabolic diseases [13]. Strategies aimed at maintaining mitochondrial health, such as caloric restriction, have been shown to extend lifespan in lower eukaryotes and mice. They also extend health span in all organisms tested including primates and delay the onset of age‐related diseases in model organisms [36, 37]. Consequently, understanding the intricate relationship between mitochondria and aging is not only crucial for unraveling the mysteries of the aging process but also for developing interventions to promote healthy aging and improve overall longevity [13, 24, 28, 38].

The link between mitochondria and cancer extends beyond energy metabolism, encompassing aspects of cell survival, proliferation, resistance to apoptosis, and shaping the tumor microenvironment. Mitochondria, through their dynamic and adaptable nature, contribute to this heterogeneity by influencing cellular functions that drive tumor progression [13, 14]. Mitochondria not only supply the essential energy needed for unlimited cell growth and division but also play a role in reconfiguring cellular processes typical of cancer cells. One of the hallmarks of cancer is altered energy metabolism, also known as the Warburg effect [39, 40]. This refers to cancer cells' preference for aerobic glycolysis, a less efficient but faster way to generate energy compared to OXPHOS in mitochondria [41, 42]. This metabolic shift enables cancer cells to fulfill the high demands for rapid proliferation and biomass production [42]. Notably, glycolysis can also occur without oxygen, which benefits fast‐growing tumors that create an anoxic environment due to insufficient vascularization. In these hypoxic conditions, glycolysis provides a continuous energy supply, supporting cancer cell survival and growth. This adaptation demonstrates how many cancer cell types rely on glycolysis for energy production and biomass synthesis, allowing them to thrive even in low‐oxygen environments. Moreover, the secretion of lactate by tumor cells plays a critical role in shaping the tumor microenvironment, further promoting cancer cell proliferation and survival. An alternative hypothesis suggests that immune cells, rather than cancer cells, maybe the primary consumers of glucose within the tumor microenvironment. This is supported by findings that myeloid cells exhibit the greatest capacity for glucose uptake, followed by T cells, while cancer cells predominantly rely on glutamine metabolism [43].

Interestingly, mitochondria in cancer cells remain functional despite changes in their behavior, structure, and energy production processes, indicating that their role extends beyond energy production. Mitochondria support cancer cell survival by maintaining mitochondrial membrane potential and releasing pro‐survival factors, which help evade programmed cell death [13, 14, 25]. Additionally, mitochondrial DNA mutations and oxidative stress contribute to genomic instability, a key factor in cancer progression [24, 38]. Recent studies have highlighted mitochondrial heterogeneity within tumors, revealing that different subpopulations of cancer cells possess unique mitochondrial characteristics [44]. This variability is linked to differences in metastatic potential, resistance to therapy, and overall prognosis. Understanding the complex relationship between mitochondria and cancer is essential for developing targeted therapies that leverage mitochondrial vulnerabilities in cancer cells [13, 14, 44]. Further investigation into the specific mechanisms by which mitochondria influence cancer biology holds promise for improving cancer treatment strategies.

Exploring the role of FAH superfamily proteins in mitochondrial function could reveal new regulatory mechanisms or therapeutic targets, given the importance of mitochondria in cancer metabolism and apoptosis. Members of the FAH superfamily, such as FAHD1, are involved in mitochondrial metabolism, and their dysregulation might affect both metabolic and apoptotic pathways in cancer cells. Investigating these connections could provide valuable insights into targeting mitochondrial vulnerabilities in cancer therapy.

## Mitochondrial metabolism and the TCA cycle

The process of OXPHOS occurs within the mitochondrial matrix and the inner mitochondrial membrane, where transporter protein complexes facilitate electron transfer through the *electron transport chain* (ETC) [45]. As electrons move along this system, protons are pumped across the inner membrane, creating an electrochemical gradient. This proton gradient powers the ATP synthase enzyme, which synthesizes ATP from *adenosine diphosphate* (ADP) and inorganic phosphate. The connection between the ETC and ATP production designates mitochondria as central to cellular energy metabolism [46, 47].

Mitochondrial metabolism encompasses the metabolism of various substrates in a cyclic series of chemical reactions collectively known as the TCA cycle [48], starting with the condensation of acetyl‐CoA with oxaloacetate to form citrate. Through a series of enzymatic reactions, citrate undergoes stepwise transformations, resulting in complete oxidation of the acetyl group, releasing two molecules of carbon dioxide and transferring high‐energy electrons to electron carriers, NADH and FADH₂, that play a pivotal role in OXPHOS, the process by which ATP is generated in mitochondria.

Apart from its role in energy production, the TCA cycle also serves as a source of intermediates for biosynthetic pathways [49, 50, 51, 52]. Several key intermediates, such as citrate, 2‐oxoglutarate (2OG, α‐ketoglutarate), succinyl‐CoA, and oxaloacetate, can be diverted from the cycle to participate in the synthesis of lipids, amino acids, heme, and other essential molecules [53, 54]. These interconnected metabolic pathways allow mitochondria to act as metabolic hubs, adapting to the cellular demands for energy and providing intermediates for biosynthetic processes [48]. TCA cycle intermediates have been reported to be involved in a plethora of processes beyond energy production, including nucleic acid synthesis, amino acid metabolism, fatty acid synthesis, post‐translational modification of proteins, and alterations of the tumor microenvironment [55]. It is also important to mention cell fate regulation through epigenetic changes, where TCA metabolites play a crucial role. Certain intermediates of the TCA cycle, such as 2‐oxoglutarate, are cofactors for enzymes involved in DNA and histone demethylation, linking metabolic states to the regulation of gene expression and cell fate decisions [56, 57].

This integration of metabolic and epigenetic regulation underscores the versatility of TCA cycle intermediates in cellular functions beyond their traditional roles in energy metabolism [47, 58, 59]. One example of this is the uptake and use of lactate from better vascularized regions of nonsmall cell lung cancer lesions. Exogenous lactate is converted to pyruvate by lactate dehydrogenase. Pyruvate is then transformed into acetyl‐CoA by the pyruvate dehydrogenase complex, which enters the citric acid cycle to produce NADH and FADH_2_. These electron carriers feed into the electron transport chain, creating a proton gradient used by ATP synthase to produce ATP. These processes enable a lower consumption of glucose, which can then be used by cancer cells situated in more hypoxic areas of the tumor mass [55, 60]. Another interesting effect of extracellular lactate is its potential to promote tumor‐supporting functions of *cancer‐associated fibroblasts* (CAFs) in the tumor stroma. Lactate‐derived 2‐oxoglutarate can induce epigenetic reprogramming in mesenchymal stem cells, promoting the formation of CAFs, and supporting growth of pancreatic ductal adenocarcinoma [61]. On the other hand, lactate secretion by CAFs has been shown to support TCA cycle progression in malignant cells and to enhance the immunosuppressive phenotype of regulatory T cells in the tumor mass [62, 63].

## The pivotal role of oxaloacetate in mitochondrial metabolism

**Fig. 1:**
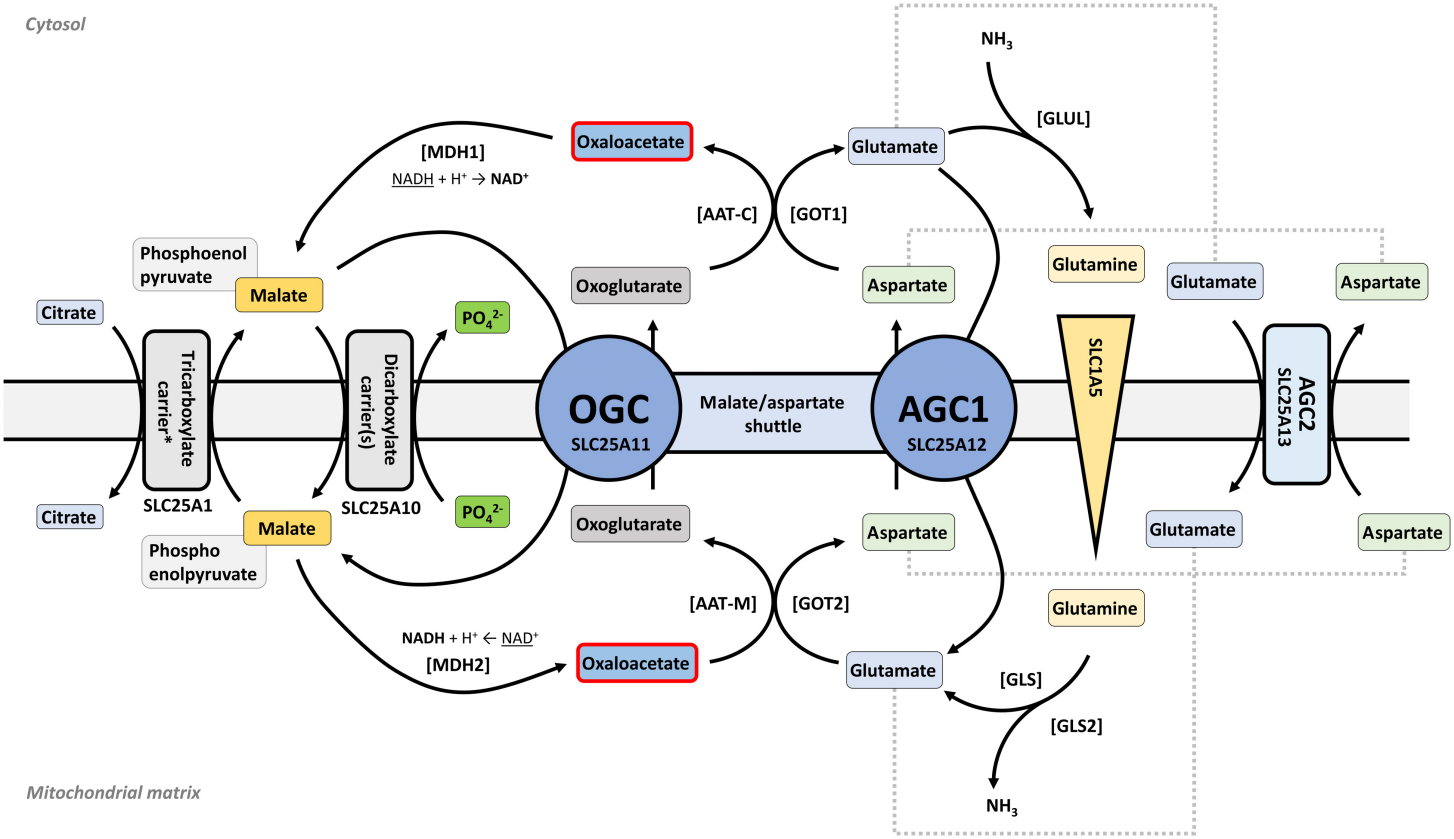
Mitochondrial and cytosolic metabolic transport pathways. The schematic depicts the transport mechanisms and metabolic pathways between the cytosol and mitochondrial matrix, emphasizing the malate/aspartate shuttle and tricarboxylate carrier systems. The figure highlights key enzymes such as *malate dehydrogenase* (MDH2 and MDH1), *glutaminase* (GLS and GLS2), and *aspartate transaminases* (GOT1 and GOT2) in the conversion and transport of metabolites including citrate, malate, oxaloacetate, aspartate, and glutamate. Notably, SLC25A1 facilitates the reversible exchange of citrate and malate, whereas phosphoenolpyruvate (PEP) can only be transported into the cytosol by this carrier. OGC, 2‐oxoglutarate carrier.

Oxaloacetate is a key metabolite in enzymatic reactions, particularly within the TCA cycle, and is pivotal for energy production and biosynthesis of amino acids, nucleotide, and many other building blocks for important cellular functions. While there are enzymes that interact with metabolites derived from oxaloacetate in various pathways, the number of enzymes directly utilizing oxaloacetate as a substrate in eukaryotic cells is limited. In what is formally known as the initial step of the TCA cycle, oxaloacetate combines with acetyl‐CoA to form citrate, which is catalyzed by the enzyme *citrate synthase* (CS) [64]. At the formal end of the TCA cycle, malate is oxidized to oxaloacetate by the enzyme *malate dehydrogenase* (MDH), generating NADH in the process. Thus, oxaloacetate regenerated in the canonical TCA cycle is again available to be condensed with another molecule of acetyl‐CoA to yield citrate, allowing the cycle to perpetuate and produce energy carriers (NADH, FADH_2_, and ATP/GTP) for the ETC and ATP synthesis. Apart from the TCA cycle, *phosphoenolpyruvate carboxykinase* (PEPCK) catalyzes the conversion of oxaloacetate to phosphoenolpyruvate, which is the starting point of gluconeogenesis, a process that synthesizes glucose from noncarbohydrate precursors. *Aspartate transaminase* (AST/SGOT) catalyzes the reversible transfer of an amino group between aspartate and 2‐oxoglutarate to form oxaloacetate and glutamate (Fig. 1).

**Fig. 2:**
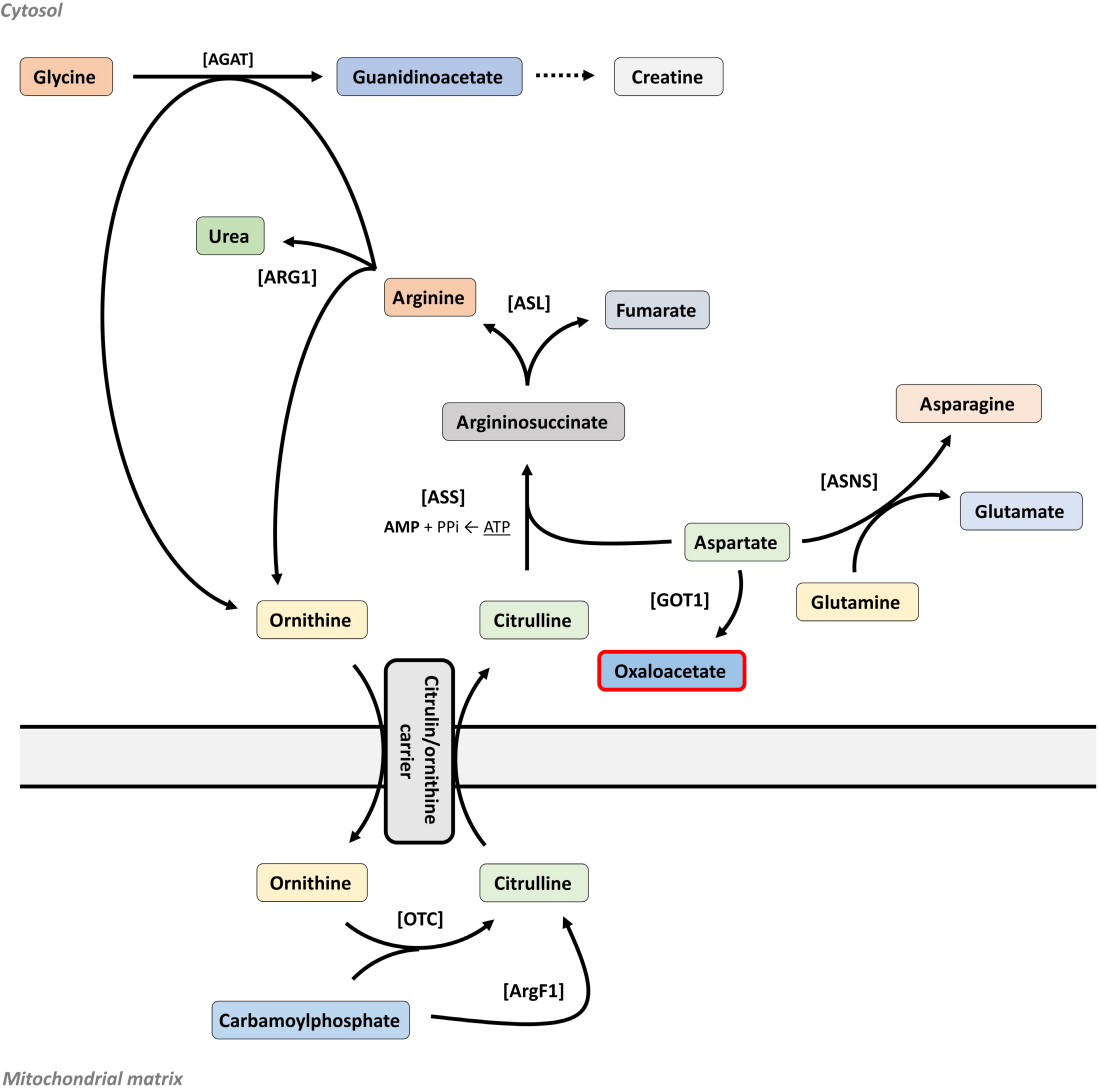
Urea cycle and aspartate–arginine metabolism. The diagram illustrates the urea cycle, focusing on the enzymatic steps converting ornithine to citrulline via *ornithine transcarbamylase* (OTC), and then to argininosuccinate via *argininosuccinate synthase* (ASS), followed by the conversion to arginine and fumarate via *argininosuccinate lyase* (ASL). The figure also includes the synthesis of creatine from glycine and guanidinoacetate, catalyzed by enzymes such as *glycine amidinotransferase* (AGAT) and *arginase* (ARG1). Aspartate and glutamine metabolism are highlighted, involving enzymes like *asparagine synthetase* (ASNS) and *glutaminase* (GLS).

Oxaloacetate also plays a role in amino acid metabolism and the urea cycle, where *aspartate aminotransferase* (AAT/SGPT) catalyzes the reversible conversion between oxaloacetate and aspartate, using 2‐oxoglutarate and glutamate as substrates (Fig. 2). The *2‐oxoglutarate carrier* (OGC) is a vital transporter protein in the inner mitochondrial membrane, essential for cellular metabolism. It facilitates the exchange of 2‐oxoglutarate with other metabolic intermediates, supporting the malate–aspartate shuttle and linking the TCA cycle to gluconeogenesis and amino acid metabolism. This exchange helps transfer reducing equivalents for ATP production and influences cellular energy homeostasis. Dysregulation of OGC can lead to metabolic imbalances and is implicated in diseases like cancer, where it contributes to metabolic reprogramming that supports rapid cell proliferation and survival. Overall, OGC is crucial for integrating and coordinating metabolic pathways essential for energy production and metabolic health.

While there is no direct enzymatic conversion between oxaloacetate and serine or cysteine, oxaloacetate plays a crucial role in metabolic pathways that indirectly support their synthesis. Serine synthesis primarily starts from 3‐phosphoglycerate, an intermediate in glycolysis [65, 66]. Cysteine synthesis involves converting serine via the trans‐sulphuration pathway, which requires homocysteine. The production of homocysteine depends on methionine availability, linked to folate metabolism [67] and the overall metabolic state of the cell, including the citric acid cycle. This illustrates the interconnected nature of metabolic pathways rather than a direct dependency.

Oxaloacetate contributes to the urea cycle by providing intermediates necessary for the synthesis of arginine [68]. This connection underscores the interconnected nature of cellular metabolism, where intermediates generated in one pathway can serve as precursors for the synthesis of molecules in other pathways, including amino acid biosynthesis [68]. As described above, oxaloacetate can be converted into aspartate through transamination with glutamate. Aspartate is a key amino acid that can be further metabolized into various other compounds necessary for cellular functions, for example, amino acids and nucleotides [69]. In the urea cycle, citrulline, formed from ornithine and carbamoyl phosphate, reacts with aspartate to form argininosuccinate in a reaction catalyzed by argininosuccinate synthase [70]. This process involves oxaloacetate, as the carbon skeleton derived from oxaloacetate is utilized in the synthesis of aspartate, which is a precursor required for arginine biosynthesis [70]. The release of fumarate derived from the cleavage of argininosuccinate provides the precursors for oxaloacetate synthesis performed by TCA cycle enzymes fumarate hydratase and MDH2. Through various enzymatic reactions, intermediates from the TCA cycle can be converted into other metabolites that participate in amino acid synthesis pathways (Fig. 1), possibly influencing the availability of precursor molecules for arginine synthesis [68, 70].

## Mitochondrial carriers and transporters related to oxaloacetate

*Mitochondrial carriers* (MCs) play a critical role in cellular metabolic homeostasis by facilitating the transport of key metabolites across mitochondrial membranes [71, 72, 73]. Despite the essential role of oxaloacetate in metabolism, specific transporters exclusively dedicated to its translocation across mitochondrial membranes have not been conclusively identified [74, 75, 76]. Instead, oxaloacetate's movement is primarily facilitated through indirect mechanisms involving other metabolite transport systems [77]. This indirect transport is integral to the malate–aspartate shuttle [78, 79], a critical system for transferring reducing equivalents between the cytosol and mitochondria. In this shuttle, oxaloacetate is reduced to malate, transported across the mitochondrial membrane, and then re‐oxidized to oxaloacetate in the cytosol (Fig. 1).

Key transporters involved in the metabolism of oxaloacetate include the *aspartate/glutamate carriers* (SLC25A12 and SLC25A13), which are vital components of the malate–aspartate shuttle [79, 80]. These carriers enable the exchange of aspartate and glutamate across the mitochondrial membrane, which can be substrates for transaminases to produce oxaloacetate and 2‐oxoglutarate, respectively. This process is crucial for maintaining the balance of NADH/NAD^+^ across the mitochondrial membrane, thereby supporting oxidative phosphorylation and ATP production. The *oxoglutarate carrier* (SLC25A11) plays a pivotal role in the exchange of cytosolic 2‐oxoglutarate for mitochondrial malate [80, 81]. This exchange helps maintain the flow of intermediates in the TCA cycle, ensuring the continuous operation of this central metabolic pathway.

Another carrier involved in the metabolism of oxaloacetate is the *citrate carrier* (SLC25A1) exporting citrate from the mitochondria into the cytosol, where it is cleaved into oxaloacetate and acetyl‐CoA [80, 81] by *citrate lyase* (ACLY) [81]. This step is significant in linking the TCA cycle with fatty acid synthesis and gluconeogenesis, underscoring oxaloacetate's role in broader metabolic processes. In addition, the *dicarboxylate carrier* (SLC25A10) facilitates the transport of malate and succinate, two dicarboxylates involved in the TCA cycle and gluconeogenesis [82]. Although its role in oxaloacetate transport is less direct than the aforementioned carriers, SLC25A10 contributes to the overall balance of TCA cycle intermediates, including oxaloacetate [82].

Other mitochondrial carriers, such as the *ADP/ATP carriers* (SLC25A4, SLC25A5, SLC25A6, SLC25A31) and *ATP‐Mg/Pi carriers* (SLC25A23, SLC25A24, SLC25A25, SLC25A41), are essential for mitochondrial function and energy metabolism but do not directly affect oxaloacetate metabolism [83, 84]. Similarly, *uncoupling proteins* (UCPs; SLC25A7, SLC25A8, SLC25A9, SLC25A14, SLC25A27, SLC25A30) [85] and the *ornithine carrier* (SLC25A15) [86, 87], primarily involved in regulating the mitochondrial proton gradient, C4 metabolites exchange, and the urea cycle have a minor role involvement in oxaloacetate's metabolic pathways. Also, the carnitine‐acylcarnitine carrier/translocase (CAC, SLC25A20) [88, 89] can be involved in the homeostasis of oxaloacetate due to its involvement in fatty acid oxidation and in the homeostasis of acyl‐carnitine/acyl‐CoA (including acetyl‐carnitine/acetyl‐CoA) pool together with the enzyme of the carnitine shuttle (CPT‐1, CPT‐2) [89] and carnitine O‐acetyltransferase (CRAT) [90].

In summary, the most relevant transporters for the metabolic role of oxaloacetate are the *aspartate/glutamate carriers* (SLC25A12 and SLC25A13), the *oxoglutarate carrier* (SLC25A11), and the *citrate carrier* (SLC25A1). The *dicarboxylate carrier* (SLC25A10) also plays a notable, though less direct, role. Other mitochondrial carriers, while crucial for broader mitochondrial and cellular functions, are less pertinent to the specific pathways involving oxaloacetate.

## The FAH superfamily of enzymes and FAHD1

The FAH superfamily, encompassing the *fumarylacetoacetate hydrolase* (FAH) family of enzymes [3, 4, 91], plays a vital role in various metabolic processes, particularly those associated with the catabolism of aromatic compounds [4]. The FAH superfamily members are characterized by a conserved domain, known as the FAH domain, which facilitates their catalytic activities [4, 91]. In prokaryotes, these enzymes are involved in the breakdown of tyrosine, phenylalanine, and other aromatic amino acids, contributing to the generation of key intermediates in cellular metabolism [4, 92, 93]. While the eponymous enzyme FAH acts as key enzyme in the breakdown of tyrosine in mammals as well, FAHD1 is primarily involved in mitochondrial function as oxaloacetate tautomerase and decarboxylase [2].

Understanding the intricacies of the FAH superfamily, including the role of FAHD1, holds promise for unraveling novel aspects of cellular metabolism and may provide novel insight into the development of therapeutic strategies for metabolic disorders and conditions associated with mitochondrial dysfunction. FAHD1 has been the subject of retrospective analysis. In a proteomic screen, FAHD1 exhibited alterations in post‐translational modification of human endothelial cells from young to senescent stages [94]. As research has unfolded, FAHD1's role in mitochondrial function and its impact on cellular metabolism have garnered attention, contributing to the understanding of intricate biochemical processes within cells. Subsequent studies revealed that FAHD1 regulates mitochondrial function and modulates oxaloacetate levels in the mitochondrial matrix [9, 10]. Oxaloacetate is a key intermediate in the TCA cycle, essential for ATP production. Reduced levels of OAA can impair the TCA cycle, affecting cellular energy production and proliferation [11, 12]. However, the regulation of OAA is more complex than simply its availability for ATP synthesis. The absence of FAHD1, which catalyzes OAA decarboxylation, leads to OAA accumulation, disrupting metabolic balance. This accumulation can inhibit enzymes like succinate dehydrogenase, slowing the TCA cycle, and reducing ATP efficiency despite higher OAA levels. Furthermore, disruptions in OAA decarboxylation affect anaplerotic and cataplerotic fluxes, impacting energy balance and cellular proliferation. Thus, FAHD1 is crucial for maintaining metabolic homeostasis and efficient ATP synthesis, and its absence leads to reduced cell proliferation due to these metabolic bottlenecks. This highlights the broader significance of FAHD1 in mitochondrial metabolism and energy regulation.

In the nematode *C. elegans*, the absence of the FAHD1 homolog fahd‐1 led to locomotion issues, along with a significant reduction in both oxygen consumption and mitochondrial membrane potential [95]. In human cells, shRNA‐mediated depletion of FAHD1 resulted in decreased cell proliferation, delayed mitochondrial electron transport, and induced cellular senescence in *human umbilical vein endothelial cells* (HUVEC) [12]. This was characterized by reduced oxygen consumption, impaired ATP‐coupled respiration, and a diminished mitochondrial membrane potential. Activation of the p53/p21 pathway led to premature senescence, without observed DNA damage or p16 pathway activation. Thus, mitochondrial dysfunction may induce cellular senescence through metabolic alterations independent of the DNA damage response pathway [12]. As will be shown in more detail below, FAHD1 functions within a complex network of enzymes, exerting regulatory control over tissue‐ and cell‐type‐specific metabolism. However, further research is needed to fully elucidate the molecular mechanisms and physiological functions of FAHD1. This work aims to summarize the current data of what is known about the role of FAHD1 in maintaining mitochondrial homeostasis, to promote cell survival during challenging conditions.

## Catalytic mechanism of FAHD1

The catalytic mechanism of FAHD1 as ODx involves a highly coordinated interaction with the magnesium cofactor [2, 96]. While it has been suggested by others that the decarboxylation activity of FAHD enzymes is merely a minor side activity [93], the central role of the magnesium cofactor in FAHD1's function indicates otherwise. The enzyme's active site initially binds oxaloacetate in the keto form [2, 96], but ODx function is dependent on the tautomerization of the bound oxaloacetate keto form to the enol form: The Mg‐bound planar form of oxaloacetate is still not competent for decarboxylation due to the orthogonality between the π‐orbitals of the enol double bond and the σ‐orbitals of the C‐C bond to be cleaved [2, 96]. The formation of the enolate intermediate and its stabilization is a prerequisite that sets the stage for the efficient release of CO₂, driven by favorable orbital interactions and electron distribution in the molecule. In detail, the magnesium ion ensures proper substrate orientation, enhancing efficient bond cleavage and product formation, indicating that the decarboxylation step [97, 98, 99] is vital in FAHD1's catalytic process [2, 96]. While the keto forms of oxaloacetate can be transformed into each other by simply rotating the C_2_–C_3_ single bond, the keto–enol tautomerization strictly requires the assistance of pocket water molecules or other cofactors [96]. The catalytic center of FAHD1 provides K123, a residue capable of deprotonating the Mg‐complexed highly acidic enol hydroxyl, which leads to magnesium‐bound ketonized oxaloacetate [2, 5, 10]. Residue Q109 stabilizes a conformation of the molecule that supports a parallel alignment of the σ‐bond to the π‐bond of the carbonyl [2, 5].

**Fig. 3:**
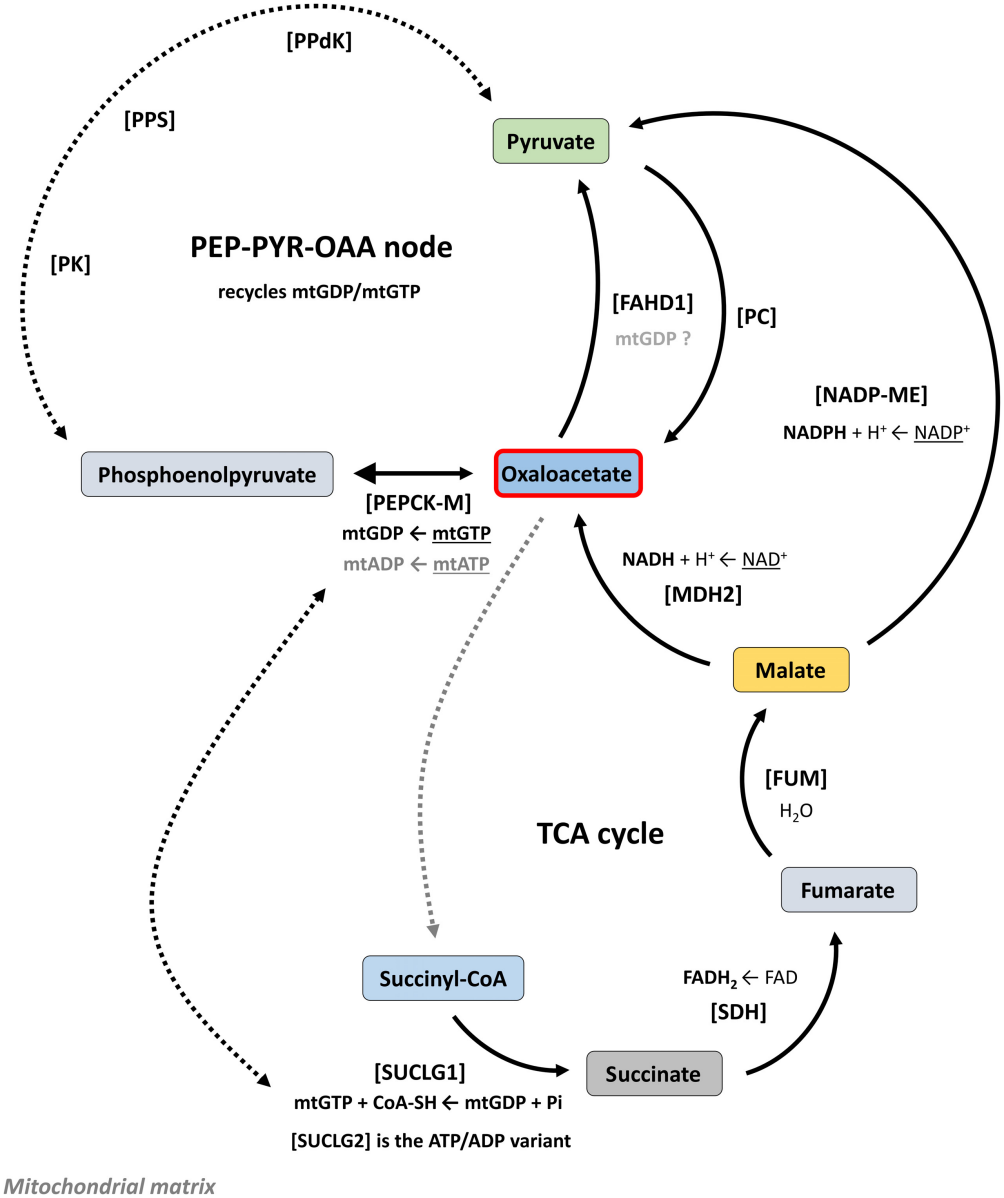
FAHD1's role in mitochondrial and cytosolic metabolic pathways. The illustration details the involvement of FAHD1 in the TCA cycle and anaplerotic reactions. Dashed lines indicate an undrawn sequence of sequential reactions. Enzymes such as *malate dehydrogenases* (MDH2 and MDH1), *pyruvate carboxylase* (PC), and mitochondrial *phosphoenolpyruvate carboxykinase* (PEPCK‐M) are shown in the conversion processes of PEP, pyruvate, and oxaloacetate. Additionally, the figure highlights the role of *succinyl‐CoA ligase* (SUCLG1 and SUCLG2) in GDP/GTP and ATP/ADP recycling within the TCA cycle.

Overall, FAHD1s Mg‐mediated decarboxylation of oxaloacetate is under strict stereo‐electronic control as direct consequence of the tautomerization process rather than a minor side activity [93], and it is highly unlikely that the enol form of oxaloacetate escapes the FAHD1 cavity without being decarboxylated. FAHD1 implicitly operates as oxaloacetate tautomerase [2] to enable its ODx function [5], and the model that incorporates both tautomerase and decarboxylase activities of FAHD1 is more aligned with the enzyme's biochemical mechanism. Numerous studies have highlighted the critical importance of decarboxylation in the function of this enzyme, underscoring its central role in the enzyme's catalytic mechanism. Furthermore, recent work suggests that FAHD1's role extends into its involvement in the PEP‐PYR‐OAA pathway, which maintains the biochemical balance between the essential metabolites phosphoenolpyruvate (PEP), pyruvate (PYR), and oxaloacetate (OAA) [10, 100]. This implies that FAHD1 may influence the metabolic flux through these key intermediates in cellular metabolism [10]. This pathway is critical in cellular metabolism, providing a second link, independent of the *pyruvate dehydrogenase* (PDH) complex, between glycolysis and the TCA cycle. Importantly, the PEPCK‐M reaction in this pathway is connected to the generation of mitochondrial GDP (mtGDP) (Fig. 3).

This nucleotide acts as a cofactor for several mitochondrial enzymes, including those in complex II. In this context, FAHD1's activity in converting oxaloacetate to pyruvate is essential for maintaining the balance of metabolic intermediates. Recent findings suggest that mitochondrial GDP may also serve as a cofactor for FAHD1 (supplementary material in Heberle *et al*. [10]). The catalytic efficiency of FAHD1 may be significantly enhanced by this intricate coordination of cofactors and substrate positioning.

In summary, FAHD1 is essential for mitochondrial metabolism due to its ODx activity [1, 5] (implicitly suggesting oxaloacetate tautomerase activity [2, 5, 96]). Beyond this, FAHD1 integrates with broader metabolic pathways, significantly affecting the generation and utilization of key metabolic intermediates and cofactors. It plays a crucial role in the conversion pathway of PEP to PYR and then to OAA, highlighting its involvement in cellular energy homeostasis [10]. Considering that the keto–enol tautomerization strictly requires the assistance of a water molecule or cofactor [96], its possible interaction with mitochondrial GDP [10] underscores its importance in maintaining and regulating metabolic processes within the cell.

## FAHD1 contributes to the regulation of glucose and glutamine metabolism

### Complex II inhibition by oxaloacetate may lead to the onset of cellular senescence

The involvement of complex II, also known as *succinate dehydrogenase* (SDH), in cellular metabolism is becoming an increasingly intriguing topic in aging biology and cancer research, particularly concerning its inhibition by oxaloacetate [101, 102, 103]. As a key component of both the TCA cycle and the mitochondrial respiratory chain, complex II has been implicated in regulating cellular senescence through multiple mechanisms [104]. Of note, premature cellular senescence caused by FAHD1 deficiency in human endothelial cells shares similarities with complex I based *mitochondrial dysfunction‐associated senescence* (MiDAS) [11, 105, 106]. Unlike replicative senescence, MiDAS cells exhibit a reduced range of SASP factors and lack DNA damage. This form of senescence can be attributed to a decreased NAD^+^/NADH ratio and reduced OXPHOS, ultimately leading to energy breakdown [11, 12, 105].

Dysregulation of complex II is also implicated in various aspects of cancer biology [104, 107, 108]. Somewhat surprisingly, complex II dysfunction can provide cancer cells with a metabolic advantage, facilitating rapid growth and survival [107, 108] by virtue of modulating the balance between OXPHOS and glycolysis, promoting the Warburg effect [109]. Mutations in genes encoding subunits of complex II were identified in various forms of cancer [110]. These mutations lead to succinate accumulation, inhibiting prolyl hydroxylases and stabilizing *hypoxia‐inducible factor 1α* (HIF1α). HIF1α activation, which induces genes associated with angiogenesis, glucose metabolism, and cell survival, creates a microenvironment conducive to tumor growth [54, 111].

### FAHD1 depletion affects cell proliferation of glutamine dependent breast cancer cells

Breast cancer tumors exhibit heterogeneity [112], combining cell types originating from different tissue compartments, which limits therapeutic options for specific cancer types. Notably, estrogen‐receptor negative tumors, such as *triple‐negative breast cancer* (TNBC) [113], show resistance to commonly used chemotherapeutics targeting hormone receptors [114]. In recent years, identifying upregulated cancer cell‐specific metabolic routes and preferred signaling pathways in TNBC has become pivotal for exploring new therapeutic approaches [113].

Given the dependence of some TNBC cell lines on glutamine consumption, targeting glutamine metabolism emerges as a promising strategy for treating aggressive breast cancer [115, 116, 117]. Compounds like BPTES [118] and compound 968 [119] (used in the clinic under the name *Telaglenastat*), which inhibit GLS activity, are currently undergoing clinical trials for TNBC treatment [115, 116]. FAHD1 has been identified as a potential target for breast cancer cells reliant on glutamine metabolism [9]. Depleting FAHD1 through siRNA‐mediated knockdown significantly hindered the proliferation of basal breast cancer cell lines, such as BT‐20, and reduced the growth of luminal cell line MCF‐7 when cultured with glutamine as the primary carbon source [9]. In MDA‐MB‐231 cells, FAHD1 knockdown led to significant reductions in glutamate and aspartate levels (unpublished data, [9]). Additionally, glycolytic flux was enhanced in FAHD1 knockdown MCF‐7 and MDA‐MB‐231 cells [9].

FAHD1 knockdown in breast cancer cells not only reduces complex II activity, likely due to oxaloacetate accumulation, but also has broader metabolic consequences [9, 11]. Lower flux through complex II decreases electron transfer efficiency by limiting ubiquinone reduction, thereby disrupting mitochondrial respiration. This has downstream effects on TCA cycle flux, impacting the cell's ability to generate energy efficiently. Furthermore, FAHD1 knockdown influences lipid transport by altering mitochondrial bioenergetics, which can disrupt membrane synthesis and fatty acid metabolism. The disruption of these pathways also affects one‐carbon metabolism [9], critical for nucleotide synthesis and methylation reactions, which are essential for cell growth and proliferation. These metabolic shifts vary depending on the availability of substrates like glucose and glutamine, highlighting the importance of FAHD1 in maintaining metabolic flexibility in breast cancer cells.

### FAHD1 depletion enhanced reprogramming capacity in MEFs

Mitochondria are integral to the metabolic transitions involved in reprogramming somatic cells into *induced pluripotent stem cells* (iPSCs), though the specific molecular mechanisms remain incompletely understood [120, 121, 122]. Reprogramming of *murine embryonic fibroblasts* (MEFs) from wild‐type and Fahd1‐knockout mice demonstrated that the depletion of FAHD1 enhances reprogramming efficiency and increases glycolytic activity compared to wild‐type MEFs [120]. This suggests that FAHD1 activity may act as a metabolic barrier to reprogramming, as its absence alleviates constraints on glycolysis. Furthermore, Fahd1 knockout iPSCs showed elevated mitochondrial DNA copy numbers and increased expression of mitochondrial biogenesis markers, reinforcing the idea that FAHD1 depletion facilitates reprogramming by inducing mitochondrial biogenesis [120]. Although no significant reduction in basal respiration was observed in Fahd1 knockout MEFs, a substantial reduction in maximal respiratory capacity indicates initial mitochondrial dysfunction [120]. This may trigger a compensatory increase in glycolysis to maintain ATP production [120]. While embryonic stem cells rely primarily on glycolytic metabolism, iPSCs require more complex metabolic remodeling, including a shift toward OXPHOS during reprogramming [120]. Fahd1 knockout iPSCs exhibited decreased respiratory capacity and mitochondrial coupling rate, highlighting the influence of FAHD1 on mitochondrial quality and function [120]. Importantly, both wild‐type and Fahd1 knockout iPSCs expressed similar pluripotency markers and retained their ability to differentiate into all three germ layers, suggesting that FAHD1 depletion does not compromise iPSC pluripotency but instead fine‐tunes their metabolic and mitochondrial state [120].

## Current knowledge and emerging hypotheses

Ongoing research into FAHD1 aims to uncover its multifaceted role in cellular metabolism and mitochondrial function. Preliminary findings suggest that FAHD1 may regulate key metabolic pathways, including glycolysis, complex II activity, and glutaminolysis. Understanding these processes could lead to better management of metabolic conditions and improved therapeutic strategies. In breast and prostate cancers, FAHD1 inhibition might alter metabolic pathways critical for cancer cell survival, making it a potential target for cancer therapy.

The regulation of FAHD1 expression across different organs, including the brain, kidney, liver, and heart, is under investigation to reveal its role in maintaining metabolic homeostasis. Additionally, modulating TCA cycle intermediates, such as oxaloacetate, might influence immune cell activation and differentiation, impacting immune responses, and memory formation. Understanding FAHD1's multifaceted roles in these metabolic pathways can provide insights into its potential implications for conditions like cancer and metabolic diseases. This drives ongoing research to unravel these complex interactions, exploring new therapeutic targets and strategies for managing metabolic disorders and improving cellular health.

**Fig. 4:**
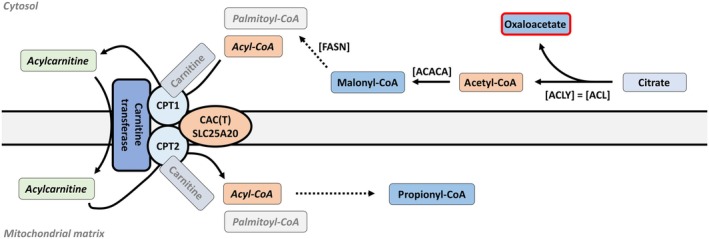
Carnitine shuttle and fatty acid β‐oxidation pathways. The carnitine shuttle system facilitates the transport of fatty acids into mitochondria for β‐oxidation. The conversion of acyl‐CoA to acylcarnitine by *carnitine palmitoyltransferase 1* (CPT1) in the cytosol and the reconversion to acyl‐CoA by CPT2 in the mitochondrial matrix are depicted. Enzymatic roles of *ATP citrate lyase* (ACLY) in citrate conversion to acetyl‐CoA, *fatty acid synthase* (FASN), and *acetyl‐CoA carboxylas*e (ACACA) in fatty acid synthesis are also highlighted.

FAHD1's impact on oxaloacetate levels highlights the interconnectedness of metabolic pathways, where changes in one intermediate can significantly influence the synthesis and regulation of essential molecules, such as fatty acids [123]. FAHD1 seems to indirectly regulate glycolysis, complex II activity, and glutaminolysis, with oxaloacetate and complex II serving as essential components of the TCA cycle. FAHD1 deficiency disrupts mitochondrial metabolism, potentially affecting heme synthesis and sterol formation. The accumulation of propionyl carnitine may indicate a disruption in carnitine usage for fatty acid trafficking, and an accumulation of propionyl carnitine in FAHD1 knockdown cells (Holzknecht *et al*.; unpublished data) (Fig. 4).

**Fig. 5:**
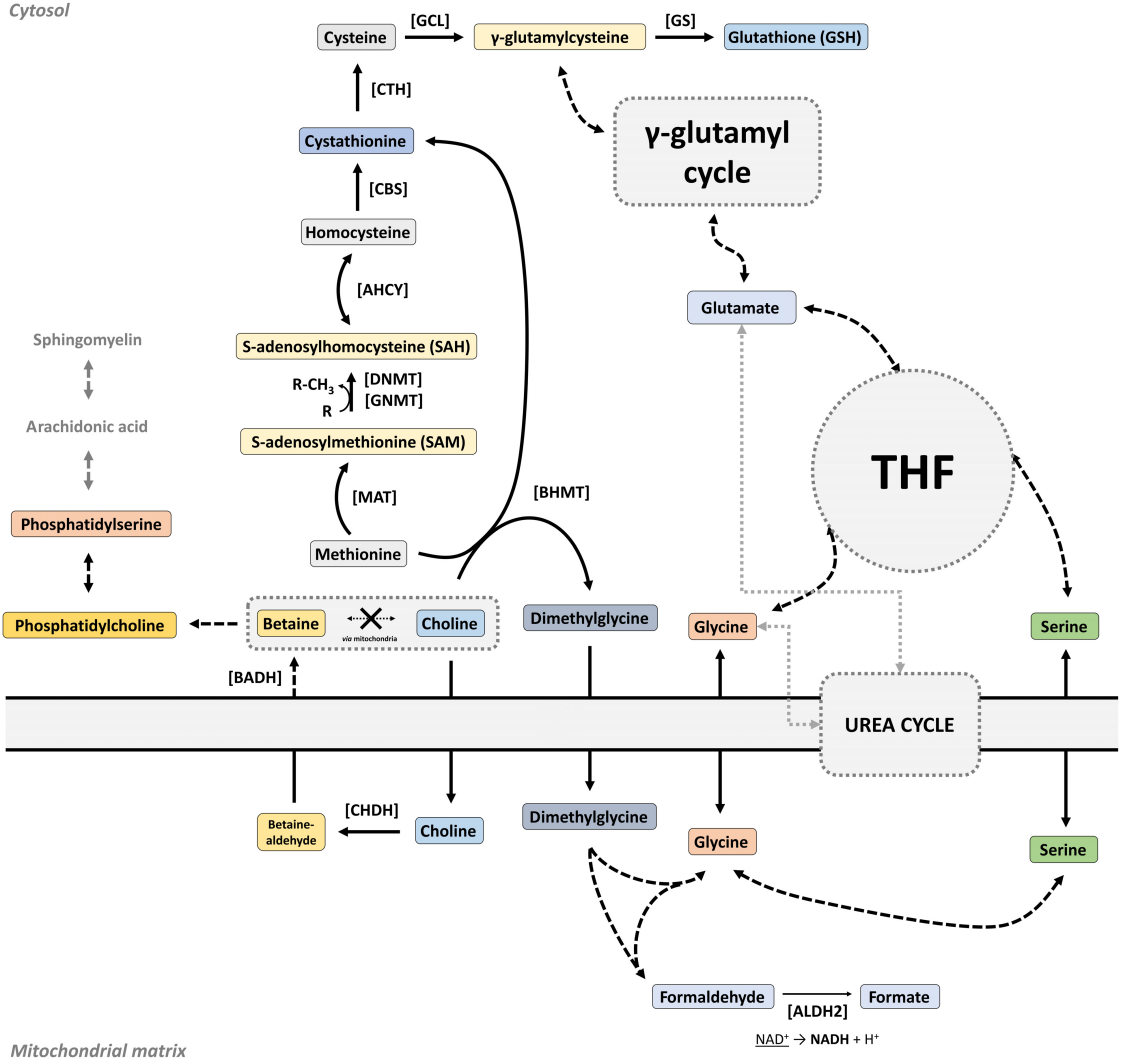
Glutathione synthesis and one‐carbon metabolism pathways. Pathways of polyamine biosynthesis and one‐carbon metabolism. The synthesis of glutathione (GSH) integrates the THF cycle, SAM/SAH cycle, and gamma‐glutamyl cycle, with serine and glycine metabolism supporting homocysteine remethylation to methionine for SAM production. Betaine from choline facilitates this process. Homocysteine is a precursor to cysteine, essential for GSH synthesis. Indirectly, oxaloacetate plays a role by participating in gluconeogenesis, affecting serine availability, and supporting the anaplerotic replenishment of TCA cycle intermediates, which are crucial for maintaining overall metabolic balance and supporting these pathways. Additionally, the arachidonic acid pathway intersects with these processes as GSH is a critical cofactor in the detoxification of reactive oxygen species generated during the metabolism of arachidonic acid, underscoring the importance of these interconnected pathways in managing oxidative stress and inflammation.

Research into FAHD1's role in cellular metabolism reveals its significant impact on mitochondrial function and broader metabolic pathways. The intricate connections between FAHD1 and the urea cycle could have broader implications for metabolic diseases and immune regulation, suggesting a possible indirect regulation of the folate cycle (up to arachidonic acid pathways) (Figs 2 and 5). Methionine, an essential amino acid in the folate cycle, serves as a precursor for S‐adenosylmethionine, a major methyl donor involved in various biosynthetic pathways. Understanding these connections could reveal new insights into metabolic regulation and gene expression. In the TCA cycle, acetyl‐CoA combines with oxaloacetate to form citrate in the mitochondria. This citrate can be exported to the cytoplasm and converted back into acetyl‐CoA by ATP citrate lyase, supporting various biosynthetic pathways, including fatty acid synthesis.

FAHD1's regulation of oxaloacetate levels plays a crucial role in maintaining the balance of intermediates necessary for lipid metabolism and energy homeostasis. In scenarios where glutamate is crucial but glutaminolysis is impaired or inefficient, several mechanisms could compensate for this necessity. For instance, enzymes involved in glutaminolysis, such as glutaminase or glutamate dehydrogenase, might be downregulated or inhibited due to genetic mutations, epigenetic modifications, destabilization of the protein, or the presence of specific inhibitors. Additionally, the availability of glutamine itself could be limited due to reduced uptake from the extracellular space or competition with other cells, particularly in nutrient‐deprived or highly competitive microenvironments such as tumors. In such cases, cells would rely on alternative sources of glutamate to fulfill their metabolic requirements. Glutamate plays a critical role in several cellular functions, including acting as a neurotransmitter, participating in the synthesis of other amino acids, and serving as a key substrate in the production of the antioxidant glutathione [124, 125]. Furthermore, glutamate is essential for the continuous activity of the malate–aspartate shuttle and the synthesis of 2‐oxoglutarate, a key intermediate in the TCA cycle, which is vital for energy production and biosynthetic processes.

Furthermore, the synthesis of compounds such as choline and betaine, which are important for cell membrane integrity and function, is linked to the folate cycle and methylation pathways. Serine and cysteine, which are interconnected through the trans‐sulphuration pathway, also play roles in these metabolic networks. The roles of choline, betaine, serine, and cysteine in maintaining membrane phospholipids and cellular methylation balance intersect with the pathway of arachidonic acid. These compounds are crucial for synthesizing and remodeling phospholipids, which are integral components of cell membranes. Although acetyl‐CoA is essential for general lipid metabolism, arachidonic acid is not synthesized directly from acetyl‐CoA. Instead, arachidonic acid is derived from linoleic acid, an essential omega‐6 fatty acid obtained through diet or recycled from membrane phospholipids. This underscores the importance of dietary intake and membrane lipid turnover in maintaining adequate levels of arachidonic acid, which is vital for producing signaling molecules like prostaglandins and leukotrienes involved in inflammation and immune responses. Furthermore, the interconnected metabolic pathways ensure that alterations in the levels of one component, such as oxaloacetate, can impact the availability and function of other critical molecules, reflecting the complex integration of cellular metabolism.

**Fig. 6:**
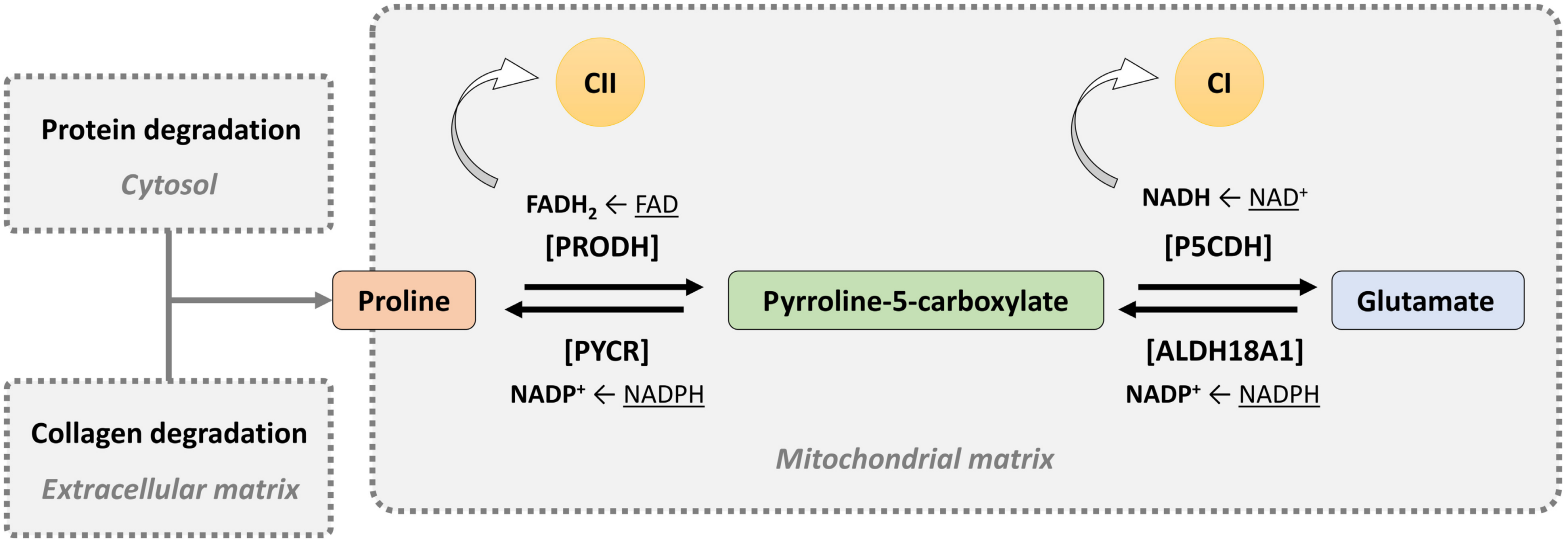
Proline metabolism and degradation pathways. Conversion of proline to pyrroline‐5‐carboxylate (P5C) by *proline dehydrogenase* (PRODH) and subsequently to glutamate via *P5C dehydrogenase* (P5CDH). The roles of enzymes such as *collagenase* and *matrix metalloproteinases* (MMPs) in the degradation of collagen and the extracellular matrix emphasize the link between protein degradation and amino acid recycling within the cytosol.

With impaired glutaminolysis, cells might upregulate pathways that provide glutamate through other means. One such pathway is the degradation of proline, particularly derived from collagen breakdown in the extracellular matrix (Fig. 6). This pathway involves the conversion of proline to pyrroline‐5‐carboxylate via proline dehydrogenase (PRODH), followed by the conversion of pyrroline‐5‐carboxylate to glutamate through pyrroline‐5‐carboxylate dehydrogenase (P5CDH). By increasing flux through this pathway, cells can sustain adequate glutamate levels to meet their metabolic demands, even when glutaminolysis is impaired. This proline‐dependent glutamate production may be especially important in cells actively remodeling their extracellular matrix, such as fibroblasts during wound healing or cancer cells undergoing metastasis. The breakdown of collagen offers a substantial source of proline, which can be redirected to fulfill the cell's need for glutamate, ensuring continued function and survival when glutaminolysis is compromised. In these contexts, FAHD1's role as a mitochondrial oxaloacetate decarboxylase becomes particularly important. Impaired glutaminolysis can lead to elevated mitochondrial oxaloacetate levels, disrupting essential metabolic processes like the TCA cycle. FAHD1 may alleviate this by reducing oxaloacetate accumulation, facilitating more efficient proline degradation to glutamate. By doing so, FAHD1 helps maintain cellular energy balance and survival in conditions of impaired glutaminolysis, contributing to processes such as tissue repair and tumor progression.

## Synopsis and outlook

The potential roles of FAHD1 in various metabolic processes and its implications for disease treatment present exciting avenues for future research. If these hypotheses are confirmed, FAHD1 could become a pivotal target for therapeutic interventions in cancer, metabolic disorders, and immune‐related diseases. Understanding the precise mechanisms by which FAHD1 regulates these pathways will provide deeper insights into cellular metabolism and its regulation. Future studies will focus on validating these findings *in vivo* and exploring the therapeutic potential of modulating FAHD1 activity. By elucidating FAHD1's functions across different tissues, targeted treatments may be developed that can address specific metabolic dysfunctions and improve overall cellular health. Future research aims to contribute significantly to the fields of metabolic regulation and disease therapy, ultimately enhancing our ability to treat complex metabolic disorders.

## Conflict of interest

The authors declare no conflict of interest.

## Author contributions

AKHW and EC conceived and authored the manuscript. MH, MP, HG, AH, YR, AS, CLP, and PJ‐D contributed specific sections and helped with the manuscript revision.
